# A telomere-to-telomere genome assembly of Chinese water deer (*Hydropotes inermis*)

**DOI:** 10.1038/s41597-026-07286-0

**Published:** 2026-04-21

**Authors:** Haotian Wu, Hengquan Zhao, Qiuting Chen, Min Chen, Ying Lu

**Affiliations:** 1 Key Laboratory of Exploration and Utilization of Aquatic Genetic Resources, Ministry of Education, Shanghai Ocean University, Shanghai, 201306 China; 2 Key Laboratory of Freshwater Aquatic Genetic Resources, Ministry of Agriculture and Rural Affairs, Shanghai Ocean University, Shanghai, 201306 China; 3 Zhejiang Zhoushan Island Ecosystem Observation and Research Station, Zhejiang Tiantong Forest Ecosystem National Observation and Research Station, Institute of Eco-Chongming, School of Ecological and Environmental Sciences, East China Normal University, Shanghai, 200241 China; 4 Hainan Institute of East China Normal University, Sanya, 572025 China

## Abstract

*Hydropotes inermis* (Chinese water deer, 2*n* = 70) is a relatively primitive small Cervidae species naturally distributed along the eastern coast of China and the Korean Peninsula. High-quality genomic resources are essential for investigating its unique adaptive and biological traits. In this study, we assembled the telomere-to-telomere (T2T) gap-free genome of a female *H. inermis* using PacBio HiFi, Oxford Nanopore Technologies (ONT), and Hi-C sequencing technologies. The assemblies were 3.45 Gb with a contig N50 of 101.09 Mb in size, which were anchored on 35 chromosomes, carrying 60 telomeres and 35 centromeres. Genome annotation annotated 33.36% repetitive sequences and 24,398 protein-coding genes. Comparative analyses with genomes of closely related species confirmed high genome integrity, continuity, and accuracy, supported by a quality value (QV) of 52.56 and a BUSCO completeness of 99.40%. This study provides a valuable genetic resource for *H. inermis* and serves as an important reference for investigating the evolutionary history of Cervidae.

## Background & Summary

The Chinese water deer (*Hydropotes inermis*) represents one of the most primitive species within the Artiodactyla family Cervidae, characterized by its small body size, absence of antlers, and well-developed upper canine teeth in males that form prominent tusks. It is primarily distributed in eastern China and the Korean Peninsula^1^. *H. inermis* exhibits several unique biological traits. For instance, it reaches sexual maturity within just 6–7 months and has the highest reproductive rate among cervids, with an average litter size of 2–3 and up to 5 offspring per birth^2^. Corresponding to its high reproductive capacity, fawns employ specialized survival strategies: newborns typically conceal themselves in dense vegetation and are nursed by the mother once every one to two days. Following nursing, the milk coagulates into curds through the action of various enzymes, enabling prolonged digestion and reducing feeding frequency^3^. As an antlerless deer, *H. inermis* holds a distinctive position in studies of cervid evolution and antler development^4^. Classical karyotypic studies have identified that the Chinese water deer possesses a diploid number of 2*n* = 70, with a predominantly acrocentric chromosome complement^5^. In recent decades, wild populations have declined sharply due to human disturbances and habitat loss, leading to its classification as a Vulnerable species on the IUCN Red List of Threatened Species^6,7^. Its adaptation to wetland plain ecosystems makes it a critical target for ecological conservation and research on reproductive and survival strategies. Moreover, *H. inermis* serves as an important model for understanding antler development and cervid husbandry. However, research on this species remains limited, and although two genome versions have been published, many genomic characteristics are still unclear.

Whole-genome sequencing is a widely used approach for deciphering complex genomic regions, comprehensively identifying structural variations, and elucidating chromosomal structure and evolution in mammals, as applied in models such as mice, sheep, and pigs. For *H. inermis*, a high-quality genome could provide insights into its unique reproductive behaviors and digestive adaptations. Nevertheless, existing genome assemblies contain numerous gaps and incomplete regions, particularly in repetitive areas like telomeres and centromeres. Telomeres, which reflect organismal health and lifespan^8^, play crucial roles in aging^9^ immunity^10^, and reproduction. Studies in humans^11^, Soay sheep^12^, and cattle bulls^13^ have demonstrated correlations between telomere length and reproductive fitness, underscoring the importance of telomere analysis in *H. inermis* given its exceptional reproductive rate. Centromeres are essential for kinetochore assembly during cell division, particularly in the rapid and stable cell divisions of early embryonic development^14^. Thus, complete centromere assembly is critical for understanding the high reproductive capacity of *H. inermis*. As of November 2025, GenBank hosts 26 chromosome-level cervid genomes spanning four subfamilies, including species such as sika deer (*Cervus nippon*), red deer (*Cervus elaphus*), white-lipped deer (*Cervus albirostris*), reindeer (*Rangifer tarandus*), white-tailed deer (*Odocoileus virginianus*), mule deer (*Odocoileus hemionus*), black muntjac (*Muntiacus crinifrons*), Reeves’s muntjac (*Muntiacus reevesi*), and Chinese water deer (*H. inermis*). However, few approach T2T standards; only four species—*Cervus nippon*^15^, *Cervus elaphus*, *Muntiacus reevesi*, and *Odocoileus virginianus*—have assemblies combining long-read sequencing and Hi-C data that approach T2T quality, and no published study has reported a complete T2T genome for any cervid.

In the present study, we used Pacific Biosciences (PacBio) HiFi sequencing, Oxford Nanopore Technologies (ONT) ultra-long sequencing, and Hi-C-assisted assembly to construct the first high-quality T2T genome of *H. inermis*. We also characterized the telomeres and centromeres of each chromosome, representing the first telomere-to-telomere genome in Cervidae. This work contributes to the conservation and evolutionary analysis of *H. inermis* and provides critical data for investigating the genetic mechanisms underlying its reproductive capacity and adaptive traits.

## Materials and Methods

### Ethics statement

The animals used in this study were approved by the ethics committee of laboratory animals of Shanghai Ocean University under the protocol number SHOU-DW-2023-216.

### Sample collection and DNA extraction

**Fig. 1:**
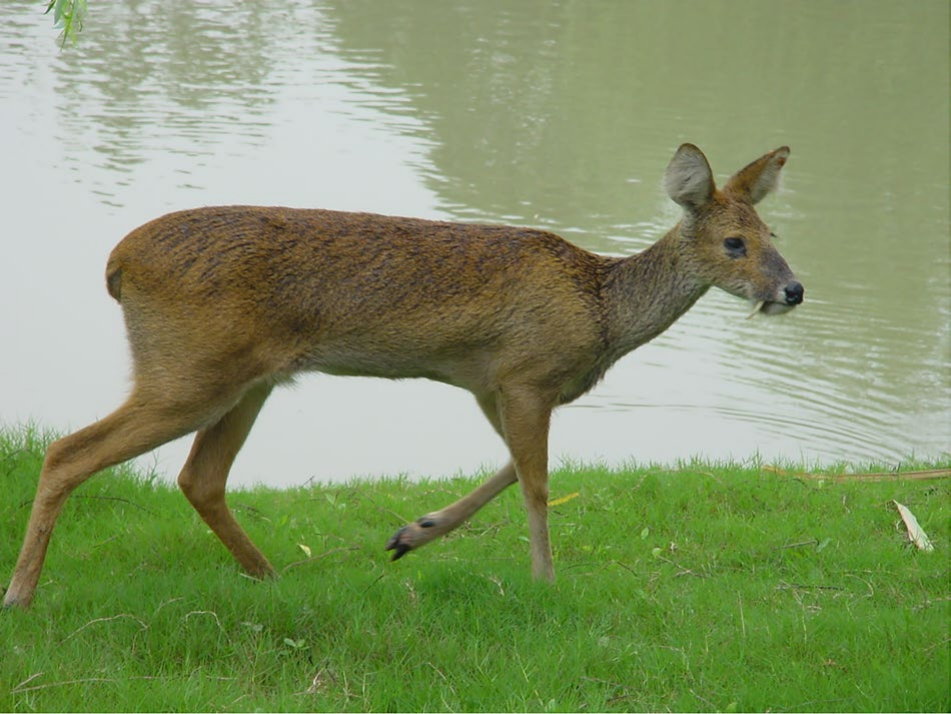
A male *H. inermis* in the Shanghai Huaxia Park, from the same population of the sequenced individual.

Muscle tissue was sampled from a 2-year-old adult female Chinese water deer (*Hydropotes inermis*, 2*n* = 70^16^) that died of natural causes (pneumonia) on June 7, 2024. The individual was housed at Shanghai Huaxia Park (Fig. 1), a government-affiliated conservation and breeding center in Shanghai, China. High-molecular-weight genomic DNA (gDNA) was isolated using the Monarch® HMW DNA Extraction Kit for Tissue (New England Biolabs, T3060) following the manufacturer’s protocol. The extraction service was provided by Biomarker Technologies Co., Ltd (Beijing, China). DNA purity was assessed with a NanoDrop spectrophotometer (Thermo Fisher Scientific, USA), and concentration was accurately quantified using a Qubit fluorometer with the dsDNA HS Assay Kit (Invitrogen, USA, Q32854).

### Library construction and sequencing

Approximately 10 µg of gDNA was sheared into ~15 kb fragments using a Megaruptor® 2 system (Diagenode). A SMRTbell library was constructed using the SMRTbell Express Template Prep Kit 2.0 (Pacific Biosciences, 100-938-900). The sheared DNA was subjected to enzymatic reactions to remove single-stranded overhangs and repair DNA damage. The DNA ends were then polished and ligated with SMRTbell adapters. Following ligation, the library was treated with exonuclease to remove unligated products and purified with 0.45X AMPure PB beads. Library size distribution and concentration were assessed using the FEMTO Pulse system (Agilent Technologies) and Qubit 3.0 Fluorometer, respectively. A size selection step targeting 15–18 kb SMRTbells was performed using the Sage ELF system (Sage Science). The final library was bound with sequencing primer and Sequel II DNA polymerase and sequenced on a PacBio Sequel II system using one 8 M SMRT Cell with the Sequel II Sequencing Kit 2.0 (Pacific Biosciences, 101-820-200), collecting 1800-minute movies. Circular consensus sequencing (CCS) reads (HiFi reads) were generated from the subread data.

For the ONT ultra-long read sequencing, high molecular-weight gDNA was size-selected (>50 kb) using the Sage HLS HMW Library System (Sage Science, USA), The qualified gDNA was then fragmented and used to construct a library with the Ultra-Long DNA Sequencing Kit (Oxford Nanopore Technologies, SQK-ULK001) according to the manufacturer’s instructions. The library was purified using magnetic beads included in the kit. Approximately 400 ng of the final library was loaded and sequenced on a PromethION R9 flow cell (Oxford Nanopore Technologies)^17^.

The Hi-C libraries were constructed to scaffold the genome to chromosome-length. Muscle tissue was cross-linked with 1% formaldehyde, quenched with 0.2 M glycine, and then lysed to isolate nuclei. Chromatin was digested with 150 U of DpnII restriction enzyme (New England Biolabs) overnight at 37 °C. The resulting sticky ends were filled in with biotin-14-dATP and ligated. After reversing cross-links by incubation with 200 µg/mL proteinase K (Thermo) at 65 °C overnight, the DNA was purified using QIAamp DNA Mini Kit (Qiagen, 51304). The purified DNA was sheared to ~400 bp fragments, and biotin-labeled ligation junctions were captured using Dynabeads MyOne Streptavidin C1 beads (Thermo Fisher Scientific). The final library was sequenced on an Illumina NovaSeq 6000 platform with a paired-end 150 bp strategy. Following the above processes, a total of 103.97 GB (~27× coverage) of ONT data with a contig N50 of 100 kb and 147.0 Gb GB (~40× coverage) of PacBio HiFi CCS data with a contig N50 of 15.31 kb were generated, as well as 380.13 GB of Hi-C data (~110× coverage) (Table 1).

**Table 1:** Statistics of the sequencing data.

Sequencing Platform	Total bases (bp)	Sequencing Depth (×)	Mean read len. (bp)	Maximum read len. (bp)	N50 read len. (bp)
ONT	103,972,763,519	27	99,950	1,218,387	100,002
PacBio HiFi	147,000,117,901	40	15,440	47,853	15,312
Hi-C	380,130,711,271	110	149.60	150	150
NGS	188,015,337,296	45	149.30	150	150
RNA-seq	40,846,245,937	—	149.20	150	150

### RNA-seq library sequencing

Total RNA was extracted from muscle tissue using the TRIzol Universal reagent (Tiangen). Poly(A)+ mRNA was enriched using oligo(dT) magnetic beads and fragmented. First-strand cDNA was synthesized using random hexamer primers, followed by second-strand synthesis. The double-stranded cDNA was purified with AMPure XP beads (Beckman Coulter). After end repair, A-tailing, and adapter ligation, the library was amplified by PCR. Library quality was assessed using a Qubit 2.0 Fluorometer and an Agilent 2100 Bioanalyzer. Quantification by qPCR ensured accurate pooling for sequencing, which was performed on an Illumina platform with a paired-end 150 bp strategy.

### Genome survey and assembly

**Fig. 2:**
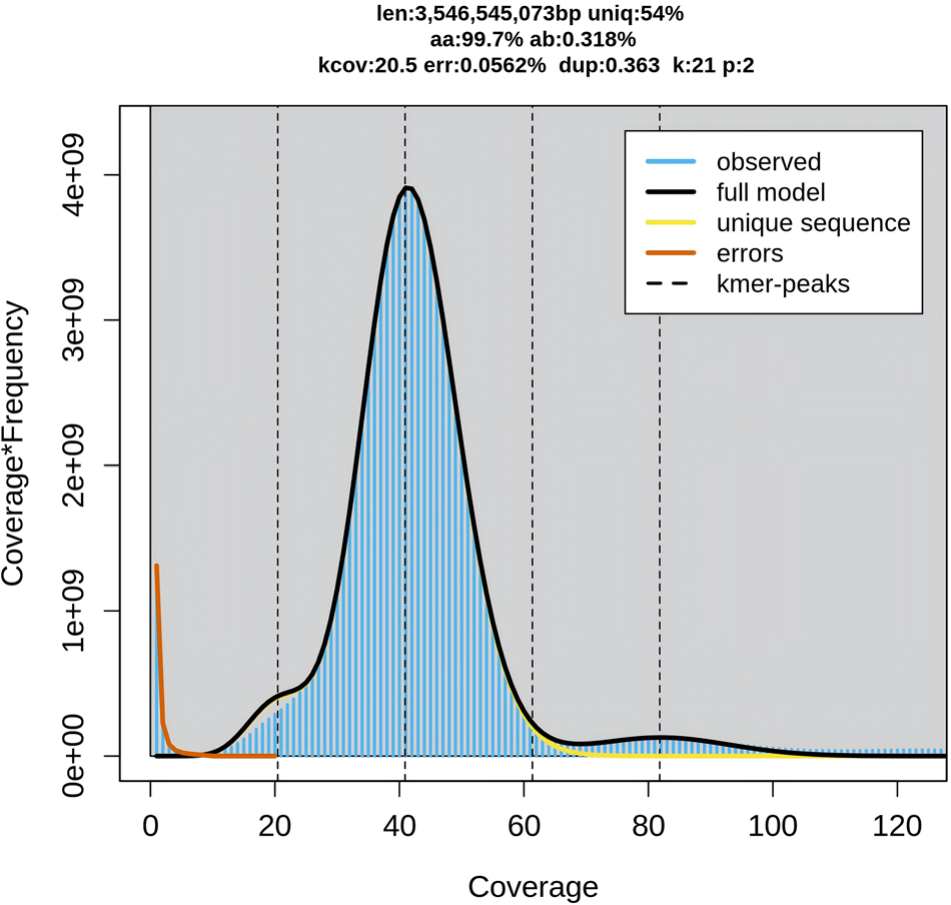
GenomeScope plots of the 21-mer frequency. The curves in black indicate the distribution of the *k*-mer frequency. The red curves near the *y*-axis show the error of the *k*-mers, which normally occur at low coverage. The estimated genome sizes (Len) and heterozygosity rates (Het) are shown at the top of each plot, as is the percentage of uniquely mapped reads (Uniq), *k*-mer coverage (Kcov), PCR error rate (Err), percentage of PCR-derived duplicates (Dup), and *k*-mer length (K).

**Fig. 3:**
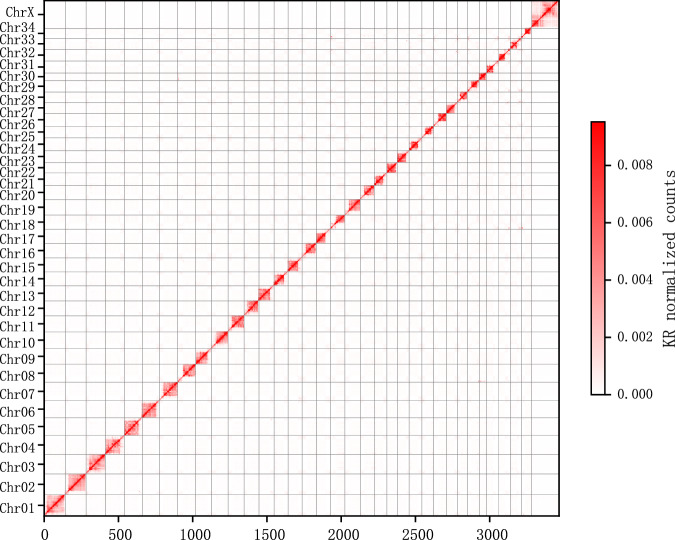
Hi-C contact heatmap under 200-Kb resolution from the female *H. inermis* sequenced in this study. Strength of intra- and inter-chromosomal interaction frequencies is indicated by a color gradient scale displayed with strong interactions in red and weaker interactions in lighter colors.

**Fig. 4:**
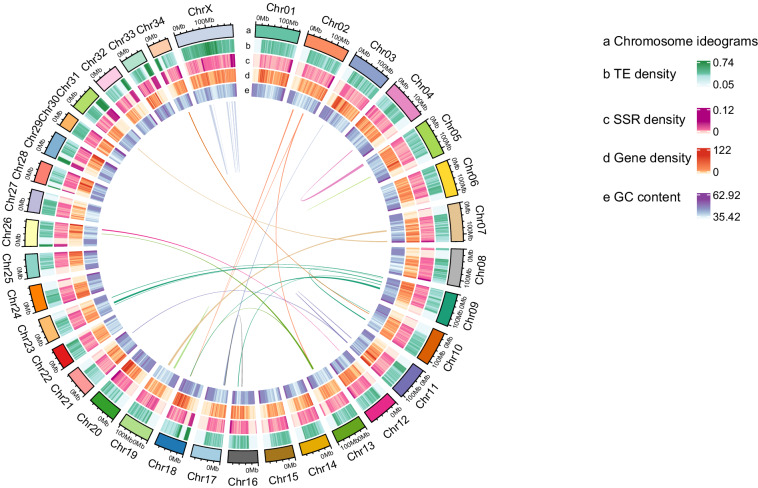
Landscape of the genome calculated in 500-kb non-overlapping windows. The circles from outside to inside: (a) Length scale of the chromosomes, (b) TE density, (c) SSR density, (d) Gene density, (e) GC content. The lines in the central area of the circles link the syntenic regions.

The genome size, heterozygosity and duplication ratio were estimated based on PacBio HiFi reads using Jellyfish (v2.3.0)^18^ and GenomeScope (v2.0)^19^. As determined from a 21-mers histogram, the estimated genome size was 3546.5 Mb, with a heterozygous ratio of 0.32% (Fig. 2). The estimated genome size was much greater than the previous assembly GCA_020226075.1 (a genome sized of 2.5 Gb) by the vast newly discovered repetitive sequences within telomeres and centromeres with PacBio HiFi long reads. A contig-level genome was assembled from the PacBio HiFi reads and ultra-long ONT reads using hifiasm (v0.19)^20^ with the parameters -t40–ul. The initial assembly was screened for contamination by aligning contigs against the NCBI GenBank nucleotide (nt), mitochondrial, and plastid databases using BLAST (v2.9.0+)^21^. The contigs identified as foreign sequences, mitochondria, or plastids were filtered out. The purified contigs were then scaffolded into chromosome-length assemblies using Hi-C data with the software Lachesis^22^ (Fig. 3). The resulting scaffolds underwent gap closing and telomere extension, utilizing the other assembly versions and long reads, to produce the final gapless T2T genome localized on 35 chromosomes (Table 2) with a genome size of 3450.42 Mb, which is highly consistent with the *k*-mer-based estimate, an N50 length of 101.09 Mb, and GC content of 39.11% (Fig. 4).

**Table 2:** Overview of the genome assemblies.

Assembled genome size (bp)	3,450,426,773
Number of Chromosomes	35
Number of gap chromosomes	0
Length of anchored chromosomes (bp)	3,450,426,773
Contig N50 Length (bp)	96,144,934
Number of contigs	35
Number of telomeres	60
Number of centromeres	35
QV	52.56
Complete BUSCO (%)	99.40

### Localization of telomeres and centromeres

**Fig. 5:**
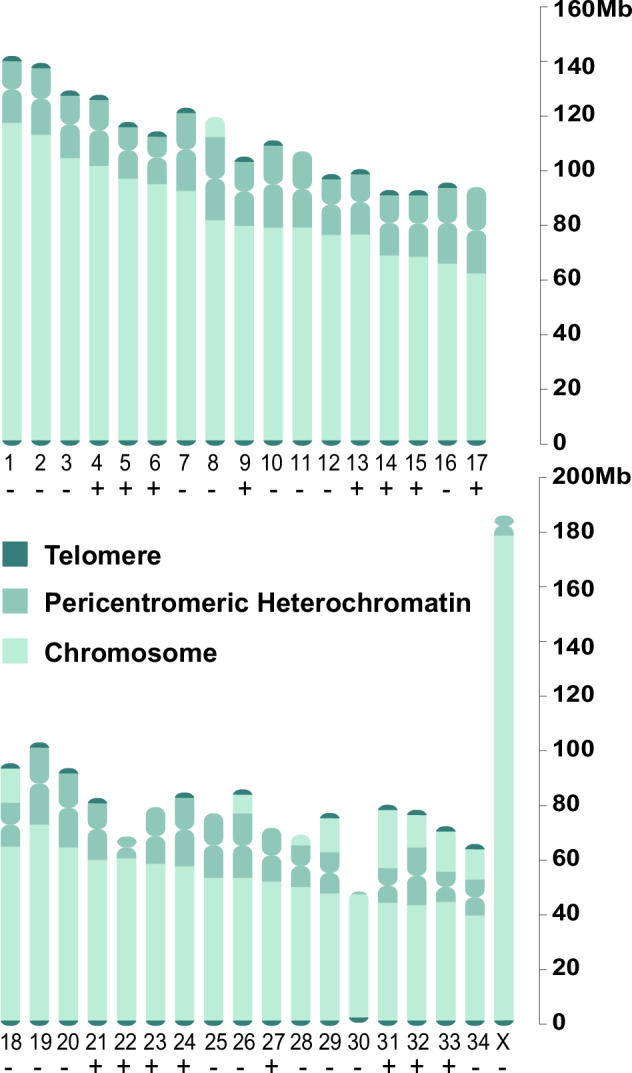
Distribution of centromere and telomeres in each chromosome scaffold of the *H. inermis* assembly.

Telomere and centromere sequences were identified to investigate genomic structure of *Hydropotes inermis*. The telomere repeat units were predicted with TIDK (v0.1.5)^23^, which determined the consensus sequences as 5′-AACCCT-3′ and 5′-AGGGTT-3′. A total of 60 telomeres were detected in the 35 chromosomes scaffolds (Table 3). Centromeric regions were characterized using Centromics (v0.3)^24^. Initially, long-read sequencing data (third-generation reads) were utilized to detect potential centromere repeats. These repeats were then aligned back to the genome assembly to obtain accurate centromere positions and sequences. Among the 35 chromosomes, the centromeric and pericentromeric heterochromatin sequences were predominantly located at the chromosomal ends. The centromere positioning is generally consistent with previous karyotypic study, as predominantly acrocentric. The centromeres had an average length of 21.59 Mb (Fig. 5) and an average GC content of 47.03%. Chinese water deer is an ancient species of the Cervidae family, characterized by over 20% centromeres and pericentromeric heterochromatin regions, which usually resulted in an under-esimation of the genome size in the *k*-mer analysis using the illumina short reads.

**Table 3:** Localization of identified telomeres and centromeres in the *H. inermis* assembly.

Chr	Chr Len. (bp)	Telomeres				Centromeres	
		5p start - end (bp)	5p len. (bp)	3p start - end (bp)	3p len. (bp)	Start - end (bp)	Len. (bp)
01	142,486,036	1–33317	33,317	142429758–142486036	56,279	33347–24392220	24,358,874
02	139,917,033	0–34764	34,765	139901126–139917013	15,888	34765–26042509	26,007,745
03	129,855,783	93–17628	17,536	129852022–129855782	3,761	33441–24701931	24,668,491
04	128,286,804	1–10911	10,911	128285213–128286050	838	102312476–128286804	25,974,329
05	118,335,903	1–1713	1,713	118334674–118335819	1,146	97660438–118319326	20,658,889
06	114,892,324	1–12204	12,204	114878568–114892324	13,757	95727766–114878567	19,150,802
07	123,510,549	0–56578	56,579	123504389–123510548	6,160	56579–30355153	30,298,575
08	120,181,151	Not detected		120169286–120181133	11,848	7346746–37752181	30,405,436
09	105,662,057	0–6696	6,697	105654058–105661956	7,899	80227035–105638305	25,411,271
10	111,594,072	0–40112	40,113	111588990–111594071	5,082	40129–31974453	31,934,325
11	107,605,195	Not detected		107594074–107605185	11,112	2–27731390	27,731,389
12	99,274,201	1–33230	33,230	99268445–99274201	5,757	33246–22278995	22,245,750
13	101,097,117	9–5720	5,712	101057202–101097116	39,915	77126322–101057199	23,930,878
14	93,439,062	10–5117	5,108	93431724–93438974	7,251	69457358–93411238	23,953,881
15	93,400,245	60–6366	6,307	93394812–93400245	5,434	69024376–93382452	24,358,077
16	96,144,934	0–19584	19,585	96135052–96144933	9,882	37058–29497914	29,460,857
17	94,525,344	1–2455	2,455	Not detected		63031266–94525344	31,494,079
18	95,967,640	18–832	815	95960040–95967639	7,600	14510361–30392012	15,881,652
19	103,620,410	1–2960	2,960	103609309–103620410	11,102	15319–30067007	30,051,689
20	94,193,941	1–14550	14,550	94184650–94193941	9,292	14551–28994731	28,980,181
21	83,252,625	40–3566	3,527	83246616–83252625	6,010	60647415–83234256	22,586,842
22	69,199,717	133–6914	6,782	Not detected		61151140–69199710	8,048,571
23	79,928,934	0–1879	1,880	Not detected		59174686–79491370	20,316,685
24	85,257,821	18–3765	3,748	85250386–85257820	7,435	58309975–85176830	26,866,856
25	77,650,735	Not detected		77645695–77650734	5,040	0–23365546	23,365,547
26	86,409,184	1–3609	3,609	86401413–86409184	7,772	8812107–32321602	23,509,496
27	72,366,311	1–7458	7,458	Not detected		52753608–71874121	19,120,514
28	69,842,294	Not detected		69834965–69842293	7,329	3833360–19025793	15,192,434
29	77,787,461	1–1672	1,672	77780911–77787442	6,532	14463758–29357440	14,893,683
30	49,035,948	Not detected		49029487–49035947	6,461	1117309–1125178	7,870
31	80,783,886	1–4798	4,798	80781276–80783886	2,611	44900414–57610761	12,710,348
32	78,943,777	1–3902	3,902	78929141–78943777	14,637	44506073–65180646	20,674,574
33	72,921,780	1–7543	7,543	72919966–72920771	806	45332929–56218804	10,885,876
34	66,426,552	0–990	991	66423141–66426551	3,411	12850711–26048597	13,197,887
X	186,629,947	Not detected		186614263–186629947	15,685	1–7356090	7,356,090

### Annotation of repetitive sequences

Repetitive sequences were annotated by integrating *de novo* prediction with homology-based approaches. *De novo* prediction was performed using RepeatModeler2 (v2.0.1)^25^, which leveraged RECON (v1.0.8)^26^ and RepeatScout (v1.0.6)^27^ to identify repetitive elements. The initial predictions were then classified using RepeatClassifier with the Dfam database (v3.5)^28^ to categorize known and novel repeats. LTR retrotransposons were specifically annotated using LTR_retriever (v2.9.0)^29^. This tool integrated outputs from LTRharvest (v1.5.10)^30^ and LTR_FINDER (v1.07)^31^ to generate comprehensive LTR element predictions. The *de novo* prediction results from RepeatModeler2 and LTR_retriever were merged with known repetitive sequences from Dfam^28^ and Repbase^32^ databases. Redundant entries were removed to construct a species-specific repeat library. The compiled repeat library was used to annotate transposable elements (TEs) across the genome using RepeatMasker (v4.1.2)^33^. This step identified and masked repetitive regions based on the custom library. The annotation results show that repetitive sequence accounts for 33.36% of the genome. Repeat sequences comprise 33.36% of the *Hydropotes inermis* genome. Among these, Long Interspersed Nuclear Elements (LINEs), Long Terminal Repeat (LTR) elements, DNA transposons, and Short Interspersed Nuclear Elements (SINEs) account for 16.40%, 7.33%, 5.56%, and 2.72%, respectively (Table 4). These proportions are notably lower than in other Cervidae species. In particular, comparison with the widely distributed red deer (*Cervus elaphus*) reveals lower repetitive content in the Chinese water deer: LINEs (16.40% vs. 37.76%), LTR retrotransposons (7.33% vs. 11.70%), and SINEs (2.72% vs. 4.22%)^34^. The distinctive repeat landscape of the *H. inermis* genome—characterized by reduced proportions of LINEs and LTRs—may reflect genomic adaptations associated with its antlerless phenotype and semi-aquatic lifestyle.

**Table 4:** Annotation of repetitive sequences.

Type	Quantity	Length of sequence (bp)	Percentage of the genome (%)
Total TEs	3,836,262	1,104,604,612	32.01
LINE	1,202,364	565,948,731	16.40
LTR	670,491	252,868,432	7.33
SINE	522,238	93,978,324	2.72
DNA	1,441,169	191,809,125	5.56
Tandem Repeats	1,241,794	39,983,513	1.15
Satellites	17,883	6,424,339	0.19
Others	1,488	237,211	0.01
Sum	5,097,427	1,151,249,675	33.36

### Prediction of protein-coding genes

**Fig. 6:**
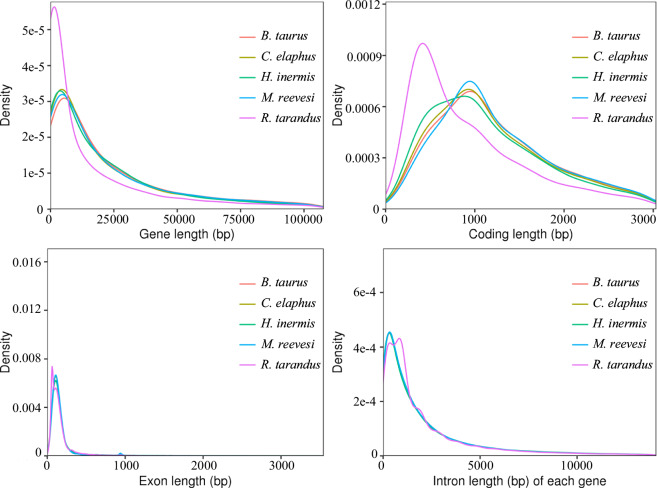
Comparison of gene structure among different bovid (*Bos taurus*) and cervid (*Cervus elaphus*, *Hydropotes inermis*, *Muntiacus reevesi*, and *Rangifer tarandus*) species, encompassing length of genes, CDSs (coding sequences), exons. and introns.

To predict protein-coding genes in the *Hydropotes inermis* genome, we employed an integrated approach combining *de novo*, homology-based, and transcriptome-based strategies. *De novo* prediction was conducted using Augustus (v3.1.0)^35^ and SNAP (2006-07-28)^36^. Homology-based prediction was performed with GeMoMa (v1.7)^37^, utilizing reference genomes from closely related species, including *Cervus elaphus*, *Bos taurus*, *Muntiacus reevesi*, and *Rangifer tarandus*. Two strategies were employed for Transcriptome-Based Prediction. First, transcripts were assembled using Hisat2 (v2.1.0)^38^ and Stringtie (v2.1.4)^39^, followed by gene prediction with GeneMarkS-T (v5.1)^40^. Second, transcripts were assembled using RNA-Bloom (v2.0.0)^41^, and gene prediction was carried out with PASA (v2.4.1)^42^. The gene predictions from the above three methods (*de novo*, homology-based, and transcriptome-based) were integrated using EVM (v1.1.1)^43^. The integrated gene set was further refined and modified with PASA (v2.4.1) to improve accuracy and completeness. Then, predicted gene sequences were functionally annotated against multiple databases, including NR, eggNOG^44^, GO, KEGG^45^, TrEMBL^46^, KOG, SWISS-PROT^46^, and Pfam^47^. This comprehensive annotation provided insights into gene functions, pathways, and protein domains. Finally, a total of 24,398 protein-coding genes were predicted, with an average gene length of 40,297.17 bp (Fig. 6). Among these, 95.03% of the genes were successfully annotated in at least one of the databases.

### Prediction of conserved non-coding RNAs

Non-coding RNAs (ncRNAs) were predicted using distinct strategies based on their structural characteristics. The software tRNAscan-SE (v1.3.1) was used to identify tRNAs, while rRNAs were primarily predicted using barrnap (v0.9). For miRNA, snoRNA, and snRNA, these ncRNAs were predicted based on the Rfam database (v14.9)^48^ and inferred using Infernal (v1.1)^49^. Overall, this annotation pipeline resulted in the prediction of 193,279 tRNAs, 546 rRNAs, and 239 miRNAs, account for 0.4% of the genome size.

## Data Records

All the sequencing data generated in this study have been deposited in the Genome Sequence Archive (GSA) in National Data Center (https://ngdc.cncb.ac.cn/gsa) under the BioProject accession PRJCA050706 and GSA accession CRA033342^50^. These include the PacBio HiFi sequencing data and the ONT ultra-long read data (FASTQ format; Run accessions CRR2303155 and CRR2303154), short-read Hi-C data and next-generation sequencing data (FASTQ format; Run accessions CRR2303157 and CRR2303156), and RNA-Seq datasets obtained from short-read sequencing (FASTQ format; Run accessions CRR2303158). These raw sequencing data have also been co-deposited in the National Center for Biotechnology Information (NCBI) Sequence Read Archive (SRA) under the accession SRP631151^51^.The final T2T gapless genome assembly of *Hydropotes inermis* is openly available in public repositories. Specifically, the assembly sequence has been deposited in the NCBI GenBank database^52^. The associated gene structure annotation files, coding sequences, and protein sequences have been deposited in Figshare^53^. All data are publicly available.

## Technical Validation

**Fig. 7:**
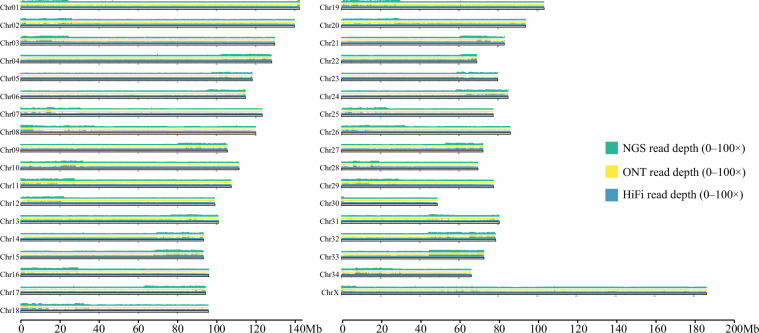
Sequencing coverage of HiFi, ONT, and NGS reads on each chromosome scaffold of the *H. inermis* assembly. Coverage depth is plotted with 50-kb windows.

The accuracy and integrity of the *Hydropotes inermis* genome assembly were rigorously validated using multiple complementary approaches. First, Hi-C analysis revealed a high degree of consistency across all chromosomes, confirming the precision of contig ordering and orientation during genome assembly. Additionally, structural integrity was demonstrated by the identification of 35 centromeres and 60 telomeres, which correspond to the expected chromosomal features in a telomere-to-telomere (T2T) assembly. To assess sequence-level accuracy, read mapping analyses were performed. Second-generation short-reads achieved a mapping rate of 99.59%, coverage of 99.96%, and an average sequencing depth of 45 × (Fig. 7). For third-generation circular consensus sequencing (CCS) reads, the mapping rate was 99.99%, with coverage of 99.98% and an average depth of 40 × . Similarly, Oxford Nanopore Technologies (ONT) long-reads exhibited a mapping rate of 98.02%, coverage of 99.96%, and an average depth of 27×. These results indicate comprehensive and reliable sequence representation across the genome. Further quality metrics supported the assembly’s robustness. The genome achieved a quality value (QV) of 52.56, reflecting high base-level accuracy. BUSCO assessment against the single-copy orthologous gene set (Artiodactyla_odb12) from OrthoDB identified 99.4% and 95.4% of the 12,594 conserved genes, underscoring the genome’s completeness and functional relevance (Table 5).Most of the used softwares were list in the (Table 6). Overall, these validation steps confirmed that the *Hydropotes inermis* genome assembly was of high quality, providing a solid foundation for studies on wetland adaptation in small cervids and reproductive biology in deer species.

**Table 5:** Completeness test of genome assemblies and annotations assessed by BUSCO.

Type	Assembly		Annotation	
	Orthologs	Percentage (%)	Orthologs	Percentage (%)
Complete BUSCOs	12517	99.4%	12,020	95.4%
Complete Single-Copy BUSCOs	12347	98.0%	11,873	94.3%
Complete Duplicated BUSCOs	170	1.3%	147	1.2%
Fragmented BUSCOs	22	0.2%	238	1.9%
Missing BUSCOs	55	0.4%	336	2.7%
Total BUSCO groups searched	12594	100%	12,594	100%

**Table 6:** Software and parameters used for genome assembly, annotation, and validation.

Software	Version	Parameter	Citation
Augustus	v3.1.0	autoAug.pl default	^35^
barrnap	v0.9	–kingdom euk–threads 1	^54^
BLAST	v2.9.0 +	default	^21^
Centromics	v0.3	default	^24^
diamond	v0.9.29.130	diamond blastp–masking 0 -e 0.001	^55^
eggnog-mapper	v1.0	emapper.py -m diamond	^56^
EVidenceModeler	v1.1.1	default	^43^
GeMoMa	v1.7	run.sh mmseqs	^37^
GenBlastA	v1.0.4	genblasta -P wublast -pg tblastn	^57^
GeneMarkS-T	v5.1	default	^40^
GeneWise	v2.4.1	genewise -both -pseudo	^58^
GenomeScope	v2.0	-k 21 -p 2	^19^
hifiasm	v0.19	default;–ul	^20^
Hisat2	v2.1.0	–dta -p 10	^38^
Infernal	v1.1	cmscan–cpu 3–rfam	^49^
InterProScan	v5.34-73.0	interproscan.sh -iprlookup -pa -f xml -dp -t p -cpu 10	^59^
Jellyfish	v2.3.0	count -C -m 21;histo -h 10000000000	^18^
Lachesis	v1	default	^22^
LTR_retriever	v2.9.0	default	^29^
PASA	v2.4.1	default	^42^
RepeatMasker	v4.1.2	repeatmasker -nolow -no_is -norna -engine wublast -parallel 8 -qq	^33^
RepeatModeler	v2.0.1	BuildDatabase -name & RepeatModeler -pa 12	^25^
SNAP	2006/7/28	default	^36^
Stringtie	v2.1.4	-p 2	^39^
TIDK	v0.1.5	default	^23^
tRNAscan-SE	v1.3.1	default	^60^

## Data Availability

All the sequencing data generated in this study have been deposited in the Genome Sequence Archive in National Data Center under the BioProject accession PRJCA050706 and GSA accession CRA033342 (https://ngdc.cncb.ac.cn/gsa/browse/CRA033342). These include PacBooio HiFi sequencing data and ONT ultra-long read data (FASTQ format; Run accessions CRR2303155 and CRR2303154), short read Hi-C data and next-generation sequencing data (FASTQ format; Run accessions CRR2303157 and CRR2303156), and RNA-Seq datasets obtained from short-read sequencing (FASTQ format; Run accessions CRR2303158). These raw sequencing data have also been co-deposited in the NCBI Sequence Read Archive under the accession SRP631151 (https://identifiers.org/ncbi/insdc.sra:SRP631151).The final T2T gapless genome assembly of *Hydropotes inermis* is openly available in public repositories. Specifically, the assembly sequence has been deposited in the NCBI GenBank database (https://identifiers.org/ncbi/insdc:JBQQXF000000000). The associated gene structure annotation files, coding sequences, and protein sequences have been deposited in Figshare (10.6084/m9.figshare.30655721.v3). All data are publicly available.

## Code availability

All commands and pipelines used in data processing were executed according to the manual and protocols of the corresponding bioinformatic software. No specific code has been developed for this study.
